# Endocytosis of glycosylphosphatidylinositol-anchored proteins

**DOI:** 10.1186/1423-0127-16-93

**Published:** 2009-10-15

**Authors:** Shaheen E Lakhan, Shefali Sabharanjak, Ananya De

**Affiliations:** 1 Global Neuroscience Initiative Foundation, Los Angeles, CA, USA

## Abstract

Glycosylphosphatidylinositol-anchored proteins (GPI-APs) represent an interesting amalgamation of the three basic kinds of cellular macromolecules viz. proteins, carbohydrates and lipids. An unusually hybrid moiety, the GPI-anchor is expressed in a diverse range of organisms from parasites to mammalian cells and serves to anchor a large number of functionally diverse proteins and has been the center of attention in scientific debate for some time now. Membrane organization of GPI-APs into laterally-organized cholesterol-sphingolipid ordered membrane domains or "rafts" and endocytosis of GPI-APs has been intensely debated. Inclusion into or exclusion from these membrane domains seems to be the critical factor in determining the endocytic mechanisms and intracellular destinations of GPI-APs. The intracellular signaling as well as endocytic trafficking of GPI-APs is critically dependent upon the cell surface organization of GPI-APs, and the associations with these lipid rafts play a vital role during these processes. The mechanism of endocytosis for GPI-APs may differ from other cellular endocytic pathways, such as those mediated by clathrin-coated pits (caveolae), and is necessary for unique biological functions. Numerous intracellular factors are involved in and regulate the endocytosis of GPI-APs, and these may be variably dependent on cell-type. The central focus of this article is to describe the significance of the endocytosis of GPI-APs on a multitude of biological processes, ranging from nutrient-uptake to more complex immune responses. Ultimately, a thorough elucidation of GPI-AP mediated signaling pathways and their regulatory elements will enhance our understanding of essential biological processes and benefit as components of disease intervention strategies.

## Background

Numerous mammalian proteins have a special posttranslational modification at their carboxy-terminal known as the glycosylphosphatidylinositol (GPI) anchor, which serves to attach the proteins to the extracellular leaflet of the cell membrane. The GPI-anchor consists of a phosphatidylinositol (PI) group attached to a carbohydrate moiety (trimannosyl-non-acetylated glucosamine), which in turn is linked through a phosphodiester bond to the carboxy-terminal amino acid of the mature protein.

GPI-anchoring of proteins is well conserved among diverse life forms, significantly involved in human diseases as malfunctions of GPI-anchored proteins (GPI-APs). Notably, defects in the enzymes of the GPI biosynthetic pathway such as phosphatidylinositol glycan class A (PIGA) and phosphatidylinositol glycan class M (PIGM) cause paroxysmal nocturnal hemoglobinuria (PNH) [1] and autosomal recessive GPI-anchor deficiency [2], respectively. PNH is characterized by deficient cell surface expression of several GPI-APs, among which are the decay-accelerating factor (DAF) and CD59. These proteins interfere with the complement system, and in their absence, patients display complement-mediated hemolysis. Similarly, in autosomal recessive GPI-anchor deficiency decreased surface expressions of CD59, CD24 among other GPI-APs on hematopoietic cells, leads to venous thrombosis and seizures. Furthermore, various GPI-APs have been implicated in human diseases such as the prion protein in several types of neurodegenerative disorders [3], fibrillin-1 in Marfan's syndrome [4], alkaline phosphatase in hypophosphatasia [5], lipoprotein lipase in chylomicronemia [6], and glypican-3 in Simpson-Golabi-Behmel syndrome [7]. Thus a clear knowledge of the functional properties of GPI-anchors is crucial for understanding protein functions.

The presence of a GPI-anchor serves the following four functional roles: (1) the apical targeting of proteins in polarized cells [8], (2) GPI-anchors mediate the cell surface organization of attached proteins through association with specialized cholesterol and sphingolipid-rich microdomains, commonly known as "lipid rafts", (3) endocytosis of GPI-APs leading to downstream signaling and, (4) cleavage of GPI-anchors by phospholipases to release soluble protein for signaling (for example, Cripto-1, [9]). In this article we describe in detail the significance of endocytosis of GPI-APs and highlight the important role of lipid rafts in the process. Existing controversies in the field and possible avenues for further research are discussed.

### Physiological Functions of GPI-APs

GPI-APs are involved in a diverse range of functions from nutrient-uptake (folate receptor (FR) [10]), complement reactions (DAF, CD59), parasite entry (CD14 in phagocytosis), cell migration and wound healing (uPAR [11]), virus receptors (folate receptor for Ebola and Marburg viruses [12]), toxin receptors (Aerolysin [13,14]), *Clostridium septicum *alpha toxin [14]) and others as elucidated below.

#### Nutrient Uptake

The human membrane folate receptor (MRF) exists in three isoforms of which the GPI-anchored α- and β-isoforms are expressed in placental tissue [10]. MFR-α is also expressed in the buccal carcinoma cell line, KB cells. Folate uptake is essential for the survival of all dividing cells. Folate is a co-factor for DNA replication enzymes as well as a substrate in thymidine synthesis. Developmental abnormalities are seen in experimental models when FR functions are obstructed with the use of antibodies or anti-sense RNA as well as in populations wherein genetic mutations in MFR-α are present [15,16]. Folate uptake mediated via GPI-anchored receptors enables cell survival in media containing low amounts of folate [17]. Chimeric FRs bearing a transmembrane protein anchor are inefficient in folate uptake and are also not subjected to the same regulatory mechanisms as the GPI-anchored form, which indicates that the GPI-anchorage directs an optimal endocytic pathway for folate absorption to be used by FR [18].

#### Neurodegenerative Prion Disease Phenotype

GPI-anchored prion proteins are the causative agents of neurodegenerative spongiform encephalopathies wherein the cellular form of the prion protein, PrP^C^, is converted to the scrapie isoforms, PrP^Sc^, resulting in the deposition of amyloid plaques on neurons [19-21]. The conversion of PrP^C ^to PrP^Sc ^occurs presumably in endocytic compartments [22,23]. Consistent with this observation, lysosomotropic agents like chloroquine as well as cysteine protease inhibitors are able to supress the conversion of PrP^C ^to PrP^Sc ^[24]. Similar to the FR, replacement of the GPI-anchor with transmembrane anchoring sequences results in reduced scrapie formation [25]. Recent hypotheses also suggest a role for prion proteins as modulators of innate as well as acquired immunity by functioning as receptors for complexes of viral RNA, viral capsid proteins and uric acid [26].

#### Toxin Receptors

Aerolysin produced by *Aeromonas hydrophila *has been shown to bind to Thy-1 [27,28], specifically to the GPI-anchor [13]. Erythrocytes from PNH patients, a disease condition where the synthesis of GPI-anchors is impaired, are resistant to intoxication by aerolysin [29]. Aerolysin is a pore-forming toxin with pore formation enhanced by the co-operative binding of about seven toxin molecules to cell membranes [30]. Oligomerization of the toxin to the heptameric form is a greatly accelerated process when the toxin is bound to the cell surface as compared to the association of the toxin molecules *in vitro *[31]. It is likely that the presence of GPI-APs in clusters at the plasma membrane is responsible for the binding and insertion of the toxin subunits in the plasma membrane [31]. Gordon et al. have demonstrated a role for FR-GPI as a receptor for the *Clostridium septicum *alpha toxin [14]. PIPLC sensitive binding of the *Helicobacter pylori *to putative GPI-anchored receptors has also been postulated [32].

#### Virus Receptors

A significant role for GPI-APs as virus receptors has emerged from several studies. FR has been identified as a receptor for filoviruses like the Ebola virus (EBV) and Marburg virus (MBV). Expression of recombinant FR in virus insensitive cell lines renders them susceptible to infection by EBV and MBV [12]. Similarly, GPI-anchored DAF has been shown to act as a co-receptor for coxsackieviruses B3 and A21 [33,34]. DAF has also been identified as the receptor for Enterovirus 70 (EV70) in leukocytes [35] and in HeLa cells [36] as well as echovirus 7 [37]. Stable transfection of NIH3T3 cells with DAF leads to binding and replication of EV70, suggesting a role for DAF in the endocytosis of EV70 [36]. Anti-DAF antibodies were effective in preventing EV70 binding to cells. In a similar manner, pretreatment of HeLa cells with PIPLC resulted in inhibition of echovirus 7 attachment to cells. Transfection of CHO cells with DAF lead to binding and infection by multiple echovirus serotypes demonstrating a role of DAF as a virus receptor [37].

#### Phagocytosis

GPI-APs are also thought to be co-receptors with CR3 in mediating non-opsonic phagocytosis of *Mycobacterium kansasii*. Antibodies against GPI-APs inhibit the non-opsonic phagocytosis of *M. kansasii *specifically. CR3 mediated phagocytosis of opsonized zymosan particles as well as opsonized *M. kansasii *is not inhibited under these conditions [38]. GPI-anchored CD55 (DAF) and CD66e (Carcinoembryonic antigen; CEA) are recruited by diffusely adhering *Escherichia coli *(Afa/Dr DAEC) strains during infection of CaCo-2 cells. The native diffuse distribution of CD55 and CD66e on the apical membrane is altered to a clustered distribution surrounding the adherent bacteria. The adhesion of bacteria can be inhibited if the interactions with these GPI-APs are prevented by pre-treatment with anti-CD55 and anti-CD66e antibodies [39]. The GPI-anchor and a short consensus region 3 (SCR3 domains) of DAF are critical for the internalization of *E. coli *expressing the Dr-adhesins [40]. GPI-anchored CD66e (also referred to as carcinoembryonic antigen, CEA) also functions as a cell surface receptor for *Neisseria gonorrhoeae *expressing the Opa^52 ^adhesin [41].

#### Lymphocyte Signaling

Signal transduction via GPI-APs is also thought to be synergistic with the activation of the T-cell receptor complex. (reviewed in [42]). Recently, stimulation of T-cells and natural killer (NK) cells by IL-18 was shown to be mediated by GPI-anchored CD48 [43]. IL-18 receptor, a complex of IL-18Rα and IL-18Rβ, binds to the protein as well as glycan anchor of GPI-anchored CD48 and thereby results in tryosine kinase activation and eventually production of interferon-gamma (IFN-gamma). These effects are prevented when cells are pretreated with phospholipase C (PIPLC) [43].

#### Other Signaling Events

Cellular prion proteins also participate in signaling events leading to the acquisition of the metastatic phenotype. In gastric cancer cell lines, PrP^c ^was found to transactivate the transcription factor MMP11 by signaling via the MEK/ERK pathway leading to enhanced metastasis *in vivo *[44].

In COS-1 cells, GPI-anchored Cripto has been shown to control the intra-endosomal sorting and trafficking of the TGF-b family member, Nodal [45]. GPI-anchored Cripto facilitates localization of Nodal (endocytosed for 40 min) to the limiting membrane of GFP-Rab4 positive endosomes. Signaling via Nodal is also facilitated by the interaction of Cripto and Nodal mediated by the EGF domain of Nodal.

These studies demonstrate that GPI-APs can participate in signaling events at the plasma membrane as well as in endosomal membranes.

GPI-APs are thus involved in various physiological processes, with the endocytosis of GPI-APs being an important regulatory mechanism in several of these processes. Clearly, in the case of the FR as well as the prion protein, access to certain endocytic compartments is mediated by the GPI-anchored isoforms of these proteins. The mechanism of intracellular trafficking might be responsible for efficient folate-uptake. It is likely that pathogens are able to exploit the normal endocytic pathways of GPI-APs to access intracellular compartments that may be distinct from the degradative lysosomal compartments. Likewise, endocytic compartments accessed by the cellular prion protein may be the sites of formation of pathogenic prion particles. In this review, we have chosen the FR, prion proteins and the urokinase type plasminogen activator receptor (uPAR) to examine the various endocytic processes that serve to internalize GPI-APs.

### Endocytosis of GPI-APs

Early studies involving endocytosis of GPI-APs [46-49] showed that endocytosis of GPI-APs could possibly occur by mechanisms distinct from those utilized by transmembrane-anchored proteins. However, these studies also involved usage of primary and secondary antibodies as ligands against GPI-APs, which was later demonstrated to be a technical error [50]. Another technique of isolation of low density membrane fractions following cold Triton-X-100 extraction has been used to "identify" membrane domains (rafts) which include GPI-APs [51]. However, the addition of cold Triton-X-100 was shown to cause the artifactual redistribution of GPI-APs in the plasma membrane of cells [52]. The endocytosis of GPI-APs should therefore be examined under conditions where these technical errors have been eliminated. GPI-anchored FR, prion proteins and uPAR have been studied extensively in order to resolve these issues.

#### Endocytosis of Folate Receptor

GPI-anchored FR was shown to recycle in cells between acid resistant (intracellular) and acid-sensitive (extracellular) pools presumably via endosomal compartments [53]. Replacement of the GPI-anchor of FR with a transmembrane one resulted in endocytosis via clathrin coated pits but the chimeric receptor was unable to deliver the endocytosed folate to the cytoplasm with the same efficiency as the GPI-anchored FR [18]. Moreover, the cytoplasmic accumulation of folate via the transmembrane anchored was not inhibited by the confluence status of the cells. The transmembrane anchored FR was therefore not subjected to cellular regulatory processes in the same manner as the native GPI-anchored FR [18]. Presumably, replacement of the GPI-anchor with a transmembrane one resulted in altered intracellular destinations of the FR where the efficiency of folate absorption was considerably reduced.

GPI-anchored FR was thought to be organized within caveolae in cholesterol-dependent clusters (detected using primary and secondary antibody staining techniques[54]) and a mechanism for folate-uptake based on this clustering was proposed (termed as "potocytosis"), whereby, transient closure and acidification of the caveolae would facilitate folate-uptake and release into the cytoplasm. This model was proposed as an explanation of the fact that FR recycles in MA104 cells [53], resulting in an intracellular and an extracellular pool. Yet these authors were not able to conclusively present an endosomal location for the intracellular pool of recycling receptors.

The potocytosis model of caveolar organization and internalization of GPI-APs were revised when it was found that cross-linking of GPI-APs lead to their selective localization into caveolae [50]. Immunodetection procedures employing primary and secondary antibodies resulted in the formation of clusters of GPI-APs in caveolae. Interestingly, the cross linking of GPI-APs is not prevented by standard paraformaldehyde based fixation techniques. The native distribution of FR visualized by labeled primary antibodies was uniform at the plasma membrane without any quantitative enrichment in any class of cell surface invaginations [50].

Cross-linking induced caveolar sequestration of GPI-APs has been confirmed in several other studies [55,56]. However, the native organization of GPI-APs has been shown to be independent of caveolae [50,57].

Studies on endocytosis of non-cross linked GPI-APs, using monovalent ligands have been possible with the FR [58,59]. Sabharanjak et al have demonstrated that FR bound by a fluorescent monovalent analogue of folic acid (PLF, [59]) is internalized into a distinct set of early endosomes that may include fluid phase markers but are distinct from the endosomes derived from the clathrin-coated pits. GPI-anchored FR is endocytosed at very early time points (typically 2 minutes) into GPI-AP enriched endosomal compartments (GEECs) that are distinct from the endosomes that contain endocytosed Transferrin receptor (TfR,[59]. These two sets of endosomes then subsequently fuse with each other and merge with the recycling endosomal compartment (REC) [58,59]. Endocytosis of FR into GEECs is also regulated by the GTPase Cdc42, whereas the endocytosis of TfR is unaffected under conditions wherein dominant negative or constitutively active Cdc42 is expressed in cells [59]. These studies have established beyond doubt that very early events in the internalization of GPI-APs like FR are completely distinct from the well characterized clathrin-coated pit mediated endocytic pathway and that GEECs represent a novel endocytic pathway.

Depletion of specific lipids like cholesterol resulted in a dramatic redirection of the FR into endosomes containing endocytosed TfR. Since the segregation of GPI-AP and transmembrane anchored proteins like TfR is evident at very early time points (2 minutes, early relative to the recycling periods of these receptors), it is clear that these sorting events are mediated at the plasma membrane. Interestingly, endocytosis via the Cdc42 regulated pathway was also sensitive to lipid perturbations, like cholesterol depletion, which altered the cell surface organization of GPI-APs in lateral inhomogeneities termed as "lipid rafts" [59,60]. These elegant experiments demonstrate that internalization of GPI-APs occurs via distinct cell surface invaginations apart from clathrin-coated pits and caveolae [59], and is dependent on lateral associations of GPI-APs with cholesterol and sphingolipids in the exoplasmic leaflet of the plasma membrane. In the end, the efficiency of folate absorption by the GPI-anchored FR is determined by these endocytic events.

Recently Chen et al [61] demonstrated that FR endosomes are delivered to endosome recycling compartment in KB and HeLa cells via microtubules. Using antibodies for dynein and kinesin I, these authors verified the role of dynein and kinesin I in traffic-mediating of FR endosomes towards the minus or plus end of microtubules, respectively. Metabolic depletion of endosomal cholesterol led to increased FR endosome motility in KB cells. This increase in FR endosome motility was proposed to be a consequence of altered associations with Rab GTPases. Cholesterol-depletion was found to lead to decreased association with Rab7 and dynein and increased association with Rab4 and kinesin (KIF3), which ultimately caused the enhanced endosome motility. These results support the earlier work by Chatterjee et al demonstrating how the depletion of cellular cholesterol and sphingolipids leads to increased recycling of GPI-APs from recycling endosomal compartments [62].

Ultimately, studies such as those detailed above help clarify the endocytosis events mediating folate-uptake and will prove valuable for the general comprehension of the efficiency of cellular folate absorption.

#### Endocytosis of Cholera Toxin B

Another piece of evidence that demonstrates that caveolin is not involved in the mechanism for internalization of GP-APs with membrane inhomogeneites is derived from studying the cholera toxin B subunit (CT-B). CT-B, which binds to the ganglioside GM1 [63], has been shown to be endocytosed via multiple endocytic pathways including caveolae (when stimulated with okadiac acid or lactosylceramide treatments) as well as a non-clathrin non-caveolae mediated endocytic pathway [64]. These CT-B positive endosomes are formed even in cells derived from caveolin-1 null mutant mice and demonstrate tubulovesicular and ring shaped morphologies similar to those of GEECs [59,64]. The ultra-structural characterization of these early endosomes shows that these are tubular structures, [65] which are capable of including cell surface bound cholera toxin, fluid phase markers like HRP and GPI-APs. In fact, in cav-1 null mouse embryonic fibroblasts, a major fraction of internalized CT-B was found to be co-localized with GPI-APs at 2 minutes post-internalization and not with transferring, a marker for the endosomes derived from clathrin-coated pits. Taken together, these results indicate that inclusion of membrane inhomogeneities consisting of GPI-APs, cholesterol, sphingolipids and GM1 can be organized in the absence of caveolin and that these structures do not utilize caveolar invaginations for endocytosis. It must be noted, however, that CT-B shows limited specificity for GM1 [66] and is capable of binding other glycolipids as well which is probably why the molecule can enter cells via multiple pathways. In order to use CT-B as a selective marker for GPI-AP associated membrane regions, a distinction between the binding sites on the plasma membrane for CT-B is essential.

#### Tubular Endosomes

Tubular endosomes have been shown to be regulated by another GTPase, ARF-6 [67,68]. ARF-6 and Tac (IL-2 receptor alpha subunit) were shown to traffic from the plasma membrane to a perinuclear endocytic compartment which was distinct from endosomes containing clathrin-coated-pit derived cargo (TfR) [67,68]. These endosomes also display tubular morphologies and were shown to facilitate endocytosis and recycling of the major histocompatibility complex class I (MHC-I) protein [69]. Naslavsky et al have proposed that the ARF-6 regulated tubular endosomes also included GPI-APs like CD59 in HeLa cells [70]. Inclusion of GPI-APs and MHC-I into the clathrin-independent tubular endosomes was also shown to be dependent upon cholesterol. Sequestration of plasma membrane cholesterol by filipin resulted in reduced endocytosis of these proteins. It is unclear whether fluid phase cargo like dextrans, representing large scale endocytosis, which is a characteristic feature of GEECs, is also included in these tubular endosomes. However, a distinction has emerged between the ARF6-regulated tubular endosomes [67,68] and GEECs from the work of Kalia et al. [71]. These researchers have shown that GEECs capable of endocytosing fluid phase markers, as well as GPI-APs, are not regulated by ARF6 in CHO cells and thus represent a completely distinct pathway. Over-expressed wild type or dominant negative ARF6 was not co-localized with GEECs and did not affect the delivery of endocytosed GPI-APs to the recycling endosomes containing TfR [71]. The acidic nature of the GEECs (pH 6.0, [71]) is also a feature that sets them apart from other endosomes like sorting endosomes, and pH neutral caveosomes [72]. Also, proteins internalized via the ARF6-regulated endocytic pathway and clathrin-mediated endocytosis do not enter into a common compartment in HeLa cells even at later time points (30 minutes) post-endocytosis [70]. Internalization of CD59 is dependent upon Rab22a activity [70], whereas the endocytosis of GPI-APs in CHO cells is regulated by Cdc42 [59]. It is therefore possible that the ARF6 regulated pathway, although cholesterol-dependent, represents a distinct pathway from that described by Sabharanjak et al in CHO cells. Alternatively, existence of multiple endocytic pathways may be a cell-type specific feature, and these studies reflect the inherent differences between HeLa (epithelial) and CHO (fibroblasts) cells in maintaining membrane flux. It would be interesting to understand whether these differences are instrumental in regulating physiological responses in various cell types. Also, the cholesterol sequestration methods used in these studies are different. Using metabolic inhibitors of cholesterol synthesis like statins is expected to reduce the overall levels of cholesterol in cells but would presumably not perturb the inherent distribution patterns of cholesterol in various cell membranes as well as in lateral membrane inhomogeneities. Filipin, on the other hand, binds to cholesterol in the plasma membrane and it is yet unclear whether this process in itself disrupts the partitioning of plasma membrane cholesterol between raft-associated and free cholesterol.

#### Endocytosis of uPAR

Urokinase type plasminogen activator (uPA) is an essential component of the plasmin generation system. Plasmin is a protease that facilitates cell mobility by digesting the integrin family of anchoring and adhesion proteins ([73], reviewed in [74]). uPA activity is in turn regulated by its inactivators, plasminogen activator inhibitors-1 and -2 (PAI-1 and -2, also referred to as serine protease inhibitors or serpins) [11]. uPA bound with PAI-1/2 or serpins is then sequestered by the GPI-anchored uPA receptor (uPAR)[11]. uPAR, thus bound to a ligand complex, has been shown to interact with the LDL-receptor related protein (LRP) [75]. Unoccupied uPAR is diffusely distributed on the cell surface, however, lateral interactions between uPAR and LRP mediated by the D3 domain of uPAR result in the inclusion of uPAR into early endosomes derived from the clathrin-coated pits [75]. The endocytosis of uPAR is thus directed by its association with another transmembrane protein. uPAR has also been shown to exist in dimer form in the basal membrane of HEK293 cells [76]. Using functional uPAR- and -EGFP or -RFP chimeras with FCS and FRET analyses, Caiolfa et al have shown that uPAR exists in monomeric as well as dimeric forms in the apical as well as the basal membrane with the dimeric form prominent in the basal membrane [76]. The dimerization is induced by the interactions of uPAR with vitronectin present in the extracellular matrix. The endocytosis of uPAR stimulated by the binding of uPA:PAI1 complex results in disengagement of the dimers into monomers that are endocytosed by binding to LRP [76]. This data re-emphasizes the effect of lateral interactions of GPI-APs with other membrane resident proteins and their influence on endocytosis of GPI-APs. Endocytosis of uPAR triggered by PAI-1 activity also results in disengagement of uPAR from interactions with fibronectin and integrins resulting in loss of cell adhesion [77]. It would be interesting to note whether other membrane lipids are instrumental in maintaining the flux of uPAR between the dimer and monomer fractions in a manner similar to that seen for raft-associated GPI-APs.

uPAR bound to uPA is known to interact with α_5_β_1 _integrin as well as EGFR and thereby facilitate signal transduction via phosphorylation of focal adhesion kinase (FAK) and EGFR (reviewed in [11] and [78]). The downstream effectors of these signaling events then facilitate cell migration [78]. Gangliosides seem to moderate these interactions in an interesting manner. Squamous carcinoma cells (SCC12), showed increased mobility in scratch as well as chemotaxis-induced migration assays post-stimulation with uPA, when these cells were depleted of gangliosides [79]. Conversely, increased levels of gangliosides GT1b and GM3 reduced the mobility of cells when challenged with uPA. Significantly, increased levels of GT1b also blocked the phosphorylation of FAK, which is normally facilitated by the interaction of uPa:uPAR with α_5_β_1 _integrin. Likewise, increased levels of GM3 inhibited phosophorylation of EGFR when cells were stimulated with uPA [79]. These results show that GT1b and GM3 are capable of disrupting the interactions of uPAR with α_5_β_1_and of the uPAR: α_5_β_1_complex with EGFR [79]. It may be envisaged that the presence of uPAR in plasma membrane inhomogeneities can be influenced by the plasma membrane levels of gangliosides. uPAR present in cholesterol-sphingolipid organized rafts would presumably be incapable of interacting with α_5_β_1 _integrin and EGFR. Thereby the rafts would serve as a regulatory sequestering mechanism that governs uPA-mediated transmembrane signaling and chemotaxis events. Alternatively, the aggregation of uPAR into rafts may also cause the endocytosis of these receptors into GEECs, or similar endosomes in other cell systems, thereby preventing uPA-induced cell motility.

**Figure 1:**
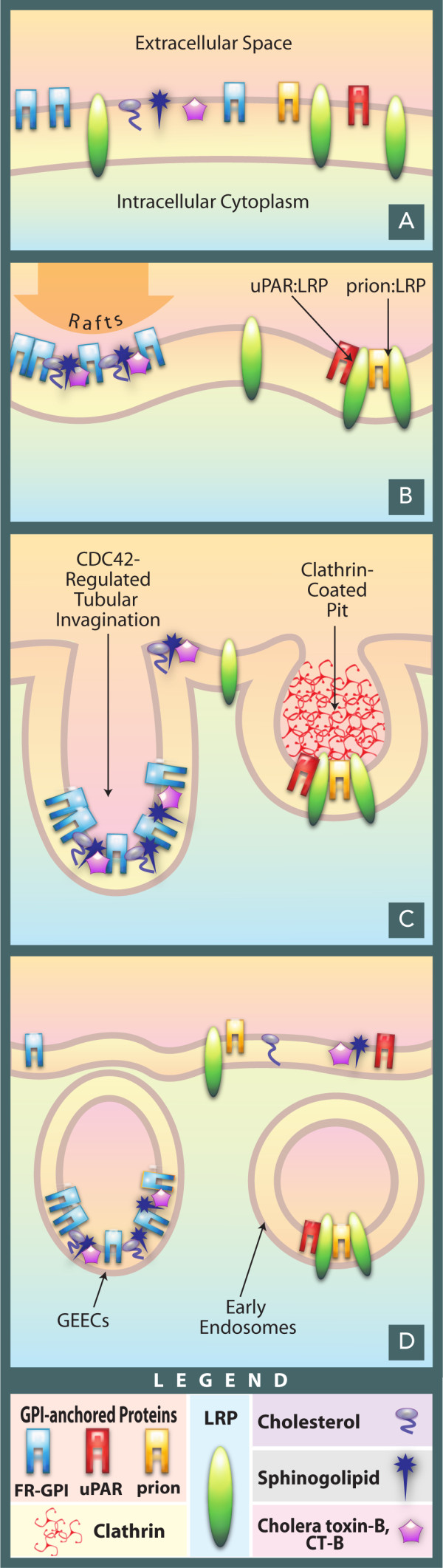
**Schematic visualization of endocytic routes adopted by various GPI-Anchored proteins and other endocytic markers**. A) Various membrane resident proteins and lipids like FR-GPI, prions, LRP, uPAR, cholesterol, sphingolipids and cholera-toxin bound to GM1 are present in a diffuse distribution in the plasma membrane. B) GPI-APs like FR-GPI align with cholesterol and sphingolipids to form lateral aggregates termed as rafts. uPAR and prion proteins, although GPI-anchored, interact with LRP and are endocytosed into the clathrin-coated pits. The interaction of the protein domains of uPAR and Prion with LRP seems to override the influence of the lateral segregation into rafts mediated by the GPI-anchor of these proteins. LRP has signal sequences in the cytoplasmic domain for recruitment into clathrin-coated pits. C and D) Raft-included markers like FR-GPI and cholera toxin bound to GM1 are endocytosed into GEECs whereas uPAR:LRP and prion;LRP complexes are endocytosed into vacuoles derived from clathrin-coated pits.

It is unclear whether uPAR can be endocytosed via GEECs (or similar CDC42-regulated endocytic pathways) when cellular levels of gangliosides, and possibly cholesterol, are elevated (see Figure 1). However the existence of such a mechanism is possible. The inclusion of GPI-APs into laterally-organized membrane rafts seems to facilitate not just endocytosis but modulation of cell surface events, like receptor engagement and signal transduction. Engagement of uPAR:uPA complexes with PAI-1 also results in disengagement of uPA:uPAR and vitronectin [77], leading to a loss of anchorage and increased mobility of cells. It is unclear whether these functions of uPAR are also modulated by cholesterol and sphingolipid levels in the plasma membrane but it would be interesting to examine these phenomena, especially in metastatic cells.

Given that cellular lipids can modulate the signaling interactions of uPA:uPAR complexes, it is likely that raft-facilitated endocytosis of uPAR may serve as spatial-temporal regulatory mechanism to control cellular signaling events.

#### Endocytosis of Prion Proteins

Cell surface resident prion proteins were shown to traffic through endocytic intermediates and this step was shown to be necessary for conversion of PrP^C ^to PrP^Sc ^[22]. In N2A neuroblastoma cells, transfected chicken PrP (chPrP) was shown to be endocytosed in endocytic structures that also showed uptake of a fluid phase marker [80]. Clathrin-coated pits were shown to be instrumental in the endocytosis of chPrP, since the endocytosis of prion proteins was shown to be inhibited by treatment with hypertonic medium [81]. This is in contradiction with the endocytic pathway demonstrated for the endocytosis of FR-GPI [59], where the fluid phase filled endosomes were shown to be distinct from those derived via clathrin-coated pits.

Prion protein endocytosis is also stimulated by binding of cupric (Cu^2+^) ions to the extracellular domain [82]. Copper-binding stimulated the endocytosis as well as recycling of GPI-anchored prion proteins for a sustained period (60 min.), indicating that perhaps the prion proteins act as receptors for Cu^2+ ^and facilitate its cellular uptake. Quite noticeably, mutant prion proteins incapable of binding copper or zinc ions are not endocytosed [83]. Mutations in the copper binding octarepeats of the sequence PHGG(G/S)WGQ results in loss of copper-binding sites as well as reduction in copper-induced endocytic trafficking of prion proteins [83].

Furthermore, endocytosis of prion proteins was found to be dependent upon dynamin and to occur independently of co-expressed GFP-GPI [84]. In co-transfected SN56 cells, dynamin K44A mutant expression reduced the endocytosis of PrP^c ^whereas GFP-GPI was endocytosed into recycling endosomes despite an over-expression of dominant-negative dynamin. Co-localization of PrP^c ^with GFP-Rab5-Q79L in early endosomes suggested that these proteins are internalized via the clathrin-coated pit-derived dynamin-dependent endocytic processes. GFP-GPI is seen to be endocytosed and trafficked to the recycling endosomes despite the expression of Rab5-Q79L. These results propose that additional factors may influence the endocytosis of PrP^C ^and the GPI-anchor itself may not be a sole deterministic factor.

The existence of a potential cell surface receptor, a 66kD protein, for PrP^c ^has been suggested from complementary hydropathy studies [85]. Recently, the LDL-receptor-related protein (LRP-1) has been shown to act as a receptor for PrP^c ^[86-88]. These results are *in concord *with the fact that cellular or over-expressed prion proteins are found in transferrin and Rab5 positive early endosomes, explaining the differences in the endocytic trafficking of other GPI-APs like FR-GPI and GFP-GPI [59,84]. However, these results contradict earlier experiments with transmembrane anchored prion proteins [25,89] wherein conversion to the infectious and neurodegerative scrapie isoform was prevented when the GPI-anchor was replaced with transmembrane anchoring sequences that directed the chimeric protein(s) to be internalized via clathrin-coated pits.

It is possible that the endocytic compartments involved in prion protein mediated copper uptake may be distinct from those that are involved in the conversion of cellular prion proteins to the scrapie isoform. Alternatively, the cell surface organization of prion proteins into cholesterol-sphingolipid domains (rafts) may influence the endocytic pathway and thereby the decision to be converted to the disease-inducing isoform(s). Since lateral interactions with LRP-1 would necessitate that prion proteins be organized in monomers (or cause a dissociation of raft included multimers), internalization into the clathrin-mediated endocytic pathway would possibly preclude lateral interactions between PrP^C ^and PrP^Sc^. It is likely that GPI-anchored prion proteins organized into lipid raft-included multimers may get internalized into acidic compartments similar to FR-GPI. These endosomes being acidic (pH ~6.0) may be the sites for the conformational changes responsible for conversion of PrP^c^into PrP^Sc ^as can be seen from *in vitro *conformational change studies [90,91]. Since protein-protein interactions between the PrP^C ^and PrP^Sc ^isoforms can possibly be facilitated by multimeric organization into cholesterol-sphingolipid ordered rafts and inclusion into acidic endosomes, it is likely that GEEC-like endosomes may be the sites for this conversion. Inclusion into the clathrin-coated pit pathway stimulated by the binding of the metal cations Cu^2+ ^and Zn^2+ ^may actually serve to be a disease preventive mechanism by promoting the dissociation of multimeric prion proteins into monomers. These hypotheses need to be evaluated *in vivo *using biophysical techniques to identify raft-associated and monomeric species of prion proteins.

A comparison of the endocytic pathway followed by PrP^C ^and PrP^Sc ^by simultaneous visualization of both proteins would probably shed some light on the endocytic destinations of these proteins. Differential regulation of endocytic pathways in various cell types can also account for the observed differences in endocytic trafficking of prion proteins. It is unclear whether all endocytic pathways remain active in all cell types, especially in terminally differentiated cell types such as neurons.

### Possible Areas for Future Research

A summary of the endocytic mechanisms of membrane markers examined in this review is presented in Table 1. Further characterization of GEECs in multiple cell types may provide some answers to the ongoing debate regarding existence of multiple endocytic pathways for GPI-APs. An existing hypothesis is that the sorting of endocytosed GPI-APs is dependent on its raft association time that is in turn determined by the raft-lipid composition. Fivaz et al have proposed a model wherein GPI-APs associated with fluid-domain preferring rafts most likely exit early endosomes quickly and are recycled, while those associated with more rigid raft domains are slowly passed into late endosomes and subsequently degraded [92]. Thus sorting fate of GPI-APs will likely be dependent on cell-type and other physiological conditions that control lipid composition of raft domains. Since endosomal systems can vary in cell types, it is problematic to arrive at a single unified pattern of trafficking of GPI-APs.

**Table 1:** Summary of endocytic routes of membrane proteins and lipids.

Protein	Invagination	Endocytic partners	Motor proteins	Cell type	Ref
**FR-GPI**	GEECs	Cholesterol and sphingolipids	CDC42	CHO cells	[59]

**chPrP**^C^	Clathrin-coated pits	LRP 1 and Cu^2+^	Dynamin	N2A	[81,84]

**Transferrin**	Clathrin-coated pits	ND	Dynamin	CHO	[59]

**GFP-GPI**	Presumably GEECs, distinct from clathrin coated pits	Presumably Cholesterol and sphingolipids	ND	SN56	[84]

**CT-B**	GEECs		Presumably CDC42	Cav-1null mouse fiborblasts	[64]

**CD59**	Tubular endosomes		ARF6 and Rab22a	HeLa	[70]

**uPAR**	Clathtrin coated pits	LRP	Dynamin	Normal Rat Kidney (NRK) and HT1080	[75]

Cellular sphingolipids and cholesterol have been shown to play a major role in the endocytic processes of GPI-APs. In human diseases wherein sphingolipid storage is aberrant concomitant redistribution cholesterol in affected cells is manifested, which in turn leads to altered GPI-AP endocytosis and distribution [93].

Recent work by Lundmark et al suggests that the protein GTPase regulator associated with focal adhesion kinase-1 (GRAF1) could be a major non-cargo marker as well as regulator for clathrin-independent endosomes such as GEECs in HeLa cells [94]. Over-expression of a dominant negative form of GRAF1 reduced the amount of GFP-GPI endocytosed in these cells. GRAF1 may be an important regulator of membrane curvature in these structures. Future analysis of such regulatory factors will provide further clarification of endocytic mechanisms controlling the endocytosis of GPI-APs and other dependent physiological events like cellular signaling.

#### Is the sorting of membrane segments mutually exclusive?

Assuming an unbiased cell surface distribution of all membrane resident proteins, it is theoretically valid to assume that LRP and LRP-1 may be included in cholesterol-sphingolipid organized rafts for some time on the cell surface. However, during internalization of these proteins as well as uPAR and prion proteins bound to them respectively, the peptide signal in the cytoplasmic tail for clathrin recruitment would be a more dominant factor than the interactions with laterally-situated cholesterol and sphingolipids. Therefore it can be safely assumed that LRP and LRP-1 cannot be endocytosed via the GEECs. Moreover, conditions that inhibit clathrin-coated pit-mediated endocytosis inhibit the endocytosis of prions (and therefore LRP-1) but not of co-transfected GFP-GPI [84].

Conversely, GPI-APs which span only the extracellular leaflet of the plasma membrane are likely to leave the rafts and can possibly be endocytosed via clathrin-coated pits. However, cellular and physiological evidence goes against this assumption. Quantification of endocytosed FR-GPI and TfR fluorescence shows that a large fraction of FR-GPI is not co-localized with TfR at 2 minutes post-internalization [59]. This ratio is significantly reduced (showing more co-localization of the two proteins) only when cellular cholesterol is depleted metabolically. Nonetheless, absorption of folate into the cytoplasm is significantly compromised in cholesterol-depleted cells, which indicates that the monomeric form or GPI-anchored folate receptor may not be the default state of these proteins in living cells [95].

Prion proteins directed towards clathrin coated pits are not capable of forming amyloid plaques whereas GPI-anchored prions do result in amyloid plaques in an endocytic compartment, sensitive to cholesterol depletion and chloroquine [25]. Likewise, the folate transport function of transmembrane-anchored FR is not regulated in the same manner to FR-GPI [18].

Although the endocytosis of monomeric GPI-APs via clathrin-coated pits cannot be ruled out absolutely with the state-of-the-art evidence, the functional correlates argue against this mechanism as being the dominant one for endocytosis of "raft-included" GPI-APs. Since cholesterol-sphingolipid ordered rafts are dynamic structures, it has been extremely difficult to establish distinct populations of raft-organized multimers and monomers of GPI-APs in living cells, visible at the resolution limit of light microscopy. That said, the differences in the morphologies of the two populations of endosomes in living cells suggest that the membrane deformation required to form the tubular GEEC invaginations can possibly be induced by the coalescence of cholesterol-sphingolipid ordered rafts. Clathrin-coated pits, on the other hand, are formed from the selective interaction of clathrin and other associated proteins with membrane segments containing membrane-spanning proteins with clathrin-recruitment signal sequences. The inherent differences in the mechanisms of these endocytic processes sufficiently indicate exclusion of cholesterol-sphingolipid ordered rafts from clathrin-coated pits and of LRP-1 from GEECs. As further support of this, Nichols [96] has demonstrated the exclusion of lipid rafts from clathrin-coated pits. It has been found that the cholesterol-dependent association of glycosphingolipid (GM1) labeled with cholera toxin B subunit did not internalize within clathrin-coated pits.

Biochemical isolation of GEECs has not been achieved yet. Membrane fragment isolation techniques are always subject to contamination from other internal membrane systems, and therefore any claims about lipid composition of selective membrane fragments becomes a controversial exercise. It may be possible to isolate GEECs by inhibiting clathrin-mediated endocytosis and then pulsing the cells with a fluorescent, non-cross-linking GPI-AP ligand for a short time to achieve selective labeling of GEECs to help isolation. However, to the best of our knowledge, this has not been achieved as yet.

## Conclusion

The significance of understanding the membrane organization and endocytic pathways of GPI-APs cannot be overstated. Recent research into the endocytosis of various GPI-APs has shown that the cell surface organization of GPI-APs into monomers or multimers may directly influence their endocytic pathways and intracellular destinations.

Since GPI-APs can participate in complex, multivalent ligand receptor interactions such as the serpin-uPA:uPAR [75,76,79], these complexes can also possibly influence the cell surface distribution and therefore perhaps the intracellular trafficking and destinations of GPI-APs. Internalization of naturally cross-linked GPI-APs (bound to ligand complexes as well as internalization partners) is a significant event in cellular physiology. However, it is essential to understand and differentiate between endocytosis of un-cross-linked and native GPI-APs in their own context. Given that cellular lipids can modulate the signaling interactions such as that of uPA:uPAR complexes, it is likely that raft-facilitated endocytosis of GPI-APs may serve as a spatial-temporal regulatory mechanism to control cellular signaling events. Furthermore, GPI-APs may display interactions which are mediated by their protein domains capable of contribute to their endocytic fate, such as uPAR and prions with LRP and LRP-1 [75,86-88]. It is imperative to understand that various GPI-APs may follow distinct endocytic mechanisms at the cell surface and endosomal pathways in various cell types and under different physiological conditions. For GPI-APs like FR and GFP-GPI, their existence into monomers or raft-ordered multimers is solely dependent upon the GPI-anchor. Although the distribution of such GPI-APs is likely to be weighted towards the raft-ordered multimers, monomeric species may be included into clathrin-coated pits. Recent research indicates the functional significance of the chosen endocytic route and demonstrates the sorting capabilities of the plasma membrane. These events may have profound effects in cellular physiology such as initiation of metastasis in cancers and onset of neurodegenerative disorders.

## Abbreviations

CEA: carcinoembryonic antigen; chPrP: chicken prion protein; CR3: Consensus region 3; CT-B: cholera toxin B subunit; DAF: decay-accelerating factor; EBV: Ebola virus; EV70: enterovirus 70; FAK: focal adhesion kinase; FR: folate receptor; GEEC: GPI-anchored protein enriched endosomal compartment; GPI: glycosylphosphatidylinositol; GPI-AP: glycosylphosphatidylinositol anchored protein; GRAF1: GTPase regulator associated with focal adhesion kinase-1; HRP: horseradish peroxidise; LRP: LDL-receptor related protein; MBV: Marburg virus; MFR: membrane folate receptor; MHC-I: major histocompatibility complex class I; NK: natural killer; PAI: plasminogen activator inhibitor; PIGA: phosphatidylinositol glycan class A; PIGM: phosphatidylinositol glycan class M; PIPLC: phospholipase C; PrPC: cellular prion protein; PrPSc: neurodegenerative scrapie isoform of prion protein; REC: recycling endosomal compartment; SCC: squamous carcinoma cell; SCR3: short consensus region 3; serpin: serine protease inhibitor; TfR: transferrin receptor; uPAR: urokinase type plasminogen activator receptor; uPA: urokinase type plasminogen activator.

## Competing interests

The authors declare that they have no competing interests.

## Authors' contributions

All authors participated in the preparation of the manuscript, and read and approved the final manuscript.

## References

[B1] Bessler M Mason PJ Hillmen P Miyata T Yamada N Takeda J Luzzatto L Kinoshita T Paroxysmal nocturnal haemoglobinuria (PNH) is caused by somatic mutations in the PIG-A gene Embo J 1994 13 110 117 8306954 10.1002/j.1460-2075.1994.tb06240.x PMC394784

[B2] Almeida AM Murakami Y Layton DM Hillmen P Sellick GS Maeda Y Richards S Patterson S Kotsianidis I Mollica L Hypomorphic promoter mutation in PIGM causes inherited glycosylphosphatidylinositol deficiency Nat Med 2006 12 846 851 10.1038/nm1410 16767100

[B3] Chesebro B Trifilo M Race R Meade-White K Teng C LaCasse R Raymond L Favara C Baron G Priola S Anchorless prion protein results in infectious amyloid disease without clinical scrapie Science 2005 308 1435 1439 10.1126/science.1110837 15933194

[B4] Robinson PN Booms P Katzke S Ladewig M Neumann L Palz M Pregla R Tiecke F Rosenberg T Mutations of FBN1 and genotype-phenotype correlations in Marfan syndrome and related fibrillinopathies Hum Mutat 2002 20 153 161 10.1002/humu.10113 12203987

[B5] Zurutuza L Muller F Gibrat JF Taillandier A Simon-Bouy B Serre JL Mornet E Correlations of genotype and phenotype in hypophosphatasia Hum Mol Genet 1999 8 1039 1046 10.1093/hmg/8.6.1039 10332035

[B6] Ameis D Kobayashi J Davis RC Ben-Zeev O Malloy MJ Kane JP Lee G Wong H Havel RJ Schotz MC Familial chylomicronemia (type I hyperlipoproteinemia) due to a single missense mutation in the lipoprotein lipase gene J Clin Invest 1991 87 1165 1170 10.1172/JCI115114 2010533 PMC295125

[B7] Veugelers M Cat BD Muyldermans SY Reekmans G Delande N Frints S Legius E Fryns JP Schrander-Stumpel C Weidle B Mutational analysis of the GPC3/GPC4 glypican gene cluster on Xq26 in patients with Simpson-Golabi-Behmel syndrome: identification of loss-of-function mutations in the GPC3 gene Hum Mol Genet 2000 9 1321 1328 10.1093/hmg/9.9.1321 10814714

[B8] Paladino S Pocard T Catino MA Zurzolo C GPI-anchored proteins are directly targeted to the apical surface in fully polarized MDCK cells J Cell Biol 2006 172 1023 1034 10.1083/jcb.200507116 16549497 PMC2063760

[B9] Watanabe K Bianco C Strizzi L Hamada S Mancino M Bailly V Mo W Wen D Miatkowski K Gonzales M Growth factor induction of Cripto-1 shedding by glycosylphosphatidylinositol-phospholipase D and enhancement of endothelial cell migration J Biol Chem 2007 282 31643 31655 10.1074/jbc.M702713200 17720976

[B10] Ross JF Chaudhuri PK Ratnam M Differential regulation of folate receptor isoforms in normal and malignant tissues in vivo and in established cell lines. Physiologic and clinical implications Cancer 1994 73 2432 2443 10.1002/1097-0142(19940501)73:9<2432::AID-CNCR2820730929>3.0.CO;2-S 7513252

[B11] Andreasen PA Egelund R Petersen HH The plasminogen activation system in tumor growth, invasion, and metastasis Cell Mol Life Sci 2000 57 25 40 10.1007/s000180050497 10949579 PMC11146824

[B12] Chan SY Empig CJ Welte FJ Speck RF Schmaljohn A Kreisberg JF Goldsmith MA Folate receptor-alpha is a cofactor for cellular entry by Marburg and Ebola viruses Cell 2001 106 117 126 10.1016/S0092-8674(01)00418-4 11461707

[B13] Diep DB Nelson KL Raja SM Pleshak EN Buckley JT Glycosylphosphatidylinositol anchors of membrane glycoproteins are binding determinants for the channel-forming toxin aerolysin J Biol Chem 1998 273 2355 2360 10.1074/jbc.273.4.2355 9442081

[B14] Gordon VM Nelson KL Buckley JT Stevens VL Tweten RK Elwood PC Leppla SH Clostridium septicum alpha toxin uses glycosylphosphatidylinositol-anchored protein receptors J Biol Chem 1999 274 27274 27280 10.1074/jbc.274.38.27274 10480947

[B15] Finnell RH Greer KA Barber RC Piedrahita JA Neural tube and craniofacial defects with special emphasis on folate pathway genes Crit Rev Oral Biol Med 1998 9 38 53 10.1177/10454411980090010201 9488247

[B16] Antony AC In utero physiology: role of folic acid in nutrient delivery and fetal development Am J Clin Nutr 2007 85 598S 603S 17284762 10.1093/ajcn/85.2.598S

[B17] Matsue H Rothberg KG Takashima A Kamen BA Anderson RG Lacey SW Folate receptor allows cells to grow in low concentrations of 5-methyltetrahydrofolate Proc Natl Acad Sci USA 1992 89 6006 6009 10.1073/pnas.89.13.6006 1631087 PMC402127

[B18] Ritter TE Fajardo O Matsue H Anderson RG Lacey SW Folate receptors targeted to clathrin-coated pits cannot regulate vitamin uptake Proc Natl Acad Sci USA 1995 92 3824 3828 10.1073/pnas.92.9.3824 7731991 PMC42054

[B19] Prusiner SB Kingsbury DT Prions--infectious pathogens causing the spongiform encephalopathies CRC Crit Rev Clin Neurobiol 1985 1 181 200 3915974

[B20] Prusiner SB Hsiao KK Human prion diseases Ann Neurol 1994 35 385 395 10.1002/ana.410350404 8154865

[B21] Prusiner SB Molecular biology and pathogenesis of prion diseases Trends Biochem Sci 1996 21 482 487 10.1016/S0968-0004(96)10063-3 9009832

[B22] Borchelt DR Taraboulos A Prusiner SB Evidence for synthesis of scrapie prion proteins in the endocytic pathway J Biol Chem 1992 267 16188 16199 1353761

[B23] Taraboulos A Raeber AJ Borchelt DR Serban D Prusiner SB Synthesis and trafficking of prion proteins in cultured cells Mol Biol Cell 1992 3 851 863 1356522 10.1091/mbc.3.8.851 PMC275644

[B24] Doh-Ura K Iwaki T Caughey B Lysosomotropic agents and cysteine protease inhibitors inhibit scrapie-associated prion protein accumulation J Virol 2000 74 4894 4897 10.1128/JVI.74.10.4894-4897.2000 10775631 PMC112015

[B25] Taraboulos A Scott M Semenov A Avrahami D Laszlo L Prusiner SB Cholesterol depletion and modification of COOH-terminal targeting sequence of the prion protein inhibit formation of the scrapie isoform J Cell Biol 1995 129 121 132 10.1083/jcb.129.1.121 7698979 PMC2120366

[B26] McBride SM Prion protein: a pattern recognition receptor for viral components and uric acid responsible for the induction of innate and adaptive immunity Med Hypotheses 2005 65 570 577 10.1016/j.mehy.2005.02.038 15913900

[B27] Abrami L Fivaz M Glauser PE Parton RG Goot FG van der A pore-forming toxin interacts with a GPI-anchored protein and causes vacuolation of the endoplasmic reticulum J Cell Biol 1998 140 525 540 10.1083/jcb.140.3.525 9456314 PMC2140172

[B28] Nelson KL Brodsky RA Buckley JT Channels formed by subnanomolar concentrations of the toxin aerolysin trigger apoptosis of T lymphomas Cell Microbiol 1999 1 69 74 10.1046/j.1462-5822.1999.00009.x 11207542

[B29] Brodsky RA Mukhina GL Nelson KL Lawrence TS Jones RJ Buckley JT Resistance of paroxysmal nocturnal hemoglobinuria cells to the glycosylphosphatidylinositol-binding toxin aerolysin Blood 1999 93 1749 1756 10029605

[B30] Goot FG van der Pattus F Wong KR Buckley JT Oligomerization of the channel-forming toxin aerolysin precedes insertion into lipid bilayers Biochemistry 1993 32 2636 2642 10.1021/bi00061a023 7680572

[B31] Abrami L Goot FG van Der Plasma membrane microdomains act as concentration platforms to facilitate intoxication by aerolysin J Cell Biol 1999 147 175 184 10.1083/jcb.147.1.175 10508864 PMC2164982

[B32] Ricci V Galmiche A Doye A Necchi V Solcia E Boquet P High cell sensitivity to Helicobacter pylori VacA toxin depends on a GPI-anchored protein and is not blocked by inhibition of the clathrin-mediated pathway of endocytosis Mol Biol Cell 2000 11 3897 3909 11071915 10.1091/mbc.11.11.3897 PMC15045

[B33] Shafren DR Dorahy DJ Ingham RA Burns GF Barry RD Coxsackievirus A21 binds to decay-accelerating factor but requires intercellular adhesion molecule 1 for cell entry J Virol 1997 71 4736 4743 9151867 10.1128/jvi.71.6.4736-4743.1997 PMC191695

[B34] Shafren DR Williams DT Barry RD A decay-accelerating factor-binding strain of coxsackievirus B3 requires the coxsackievirus-adenovirus receptor protein to mediate lytic infection of rhabdomyosarcoma cells J Virol 1997 71 9844 9848 9371658 10.1128/jvi.71.12.9844-9848.1997 PMC230302

[B35] Vuorinen T Vainionpaa R Heino J Hyypia T Enterovirus receptors and virus replication in human leukocytes J Gen Virol 1999 80 Pt 4 921 927 10211961 10.1099/0022-1317-80-4-921

[B36] Karnauchow TM Tolson DL Harrison BA Altman E Lublin DM Dimock K The HeLa cell receptor for enterovirus 70 is decay-accelerating factor (CD55) J Virol 1996 70 5143 5152 8764022 10.1128/jvi.70.8.5143-5152.1996 PMC190469

[B37] Bergelson JM Chan M Solomon KR St John NF Lin H Finberg RW Decay-accelerating factor (CD55), a glycosylphosphatidylinositol-anchored complement regulatory protein, is a receptor for several echoviruses Proc Natl Acad Sci USA 1994 91 6245 6248 10.1073/pnas.91.13.6245 7517044 PMC44175

[B38] Peyron P Bordier C N'Diaye EN Maridonneau-Parini I Nonopsonic phagocytosis of Mycobacterium kansasii by human neutrophils depends on cholesterol and is mediated by CR3 associated with glycosylphosphatidylinositol-anchored proteins J Immunol 2000 165 5186 5191 11046051 10.4049/jimmunol.165.9.5186

[B39] Guignot J Peiffer I Bernet-Camard MF Lublin DM Carnoy C Moseley SL Servin AL Recruitment of CD55 and CD66e brush border-associated glycosylphosphatidylinositol-anchored proteins by members of the Afa/Dr diffusely adhering family of Escherichia coli that infect the human polarized intestinal Caco-2/TC7 cells Infect Immun 2000 68 3554 3563 10.1128/IAI.68.6.3554-3563.2000 10816511 PMC97642

[B40] Selvarangan R Goluszko P Popov V Singhal J Pham T Lublin DM Nowicki S Nowicki B Role of decay-accelerating factor domains and anchorage in internalization of Dr-fimbriated Escherichia coli Infect Immun 2000 68 1391 1399 10.1128/IAI.68.3.1391-1399.2000 10678952 PMC97293

[B41] Gray-Owen SD Dehio C Haude A Grunert F Meyer TF CD66 carcinoembryonic antigens mediate interactions between Opa-expressing Neisseria gonorrhoeae and human polymorphonuclear phagocytes Embo J 1997 16 3435 3445 10.1093/emboj/16.12.3435 9218786 PMC1169969

[B42] Ilangumaran S He HT Hoessli DC Microdomains in lymphocyte signalling: beyond GPI-anchored proteins Immunol Today 2000 21 2 7 10.1016/S0167-5699(99)01494-2 10637551

[B43] Fukushima K Ikehara Y Yamashita K Functional role played by the glycosylphosphatidylinositol anchor glycan of CD48 in interleukin-18-induced interferon-gamma production J Biol Chem 2005 280 18056 18062 10.1074/jbc.M413297200 15760905

[B44] Pan Y Zhao L Liang J Liu J Shi Y Liu N Zhang G Jin H Gao J Xie H Cellular prion protein promotes invasion and metastasis of gastric cancer Faseb J 2006 20 1886 1888 10.1096/fj.06-6138fje 16877520

[B45] Blanchet MH Le Good JA Oorschot V Baflast S Minchiotti G Klumperman J Constam DB Cripto localizes Nodal at the limiting membrane of early endosomes Sci Signal 2008 1 ra13 10.1126/scisignal.1165027 19001664

[B46] Bamezai A Goldmacher VS Rock KL Internalization of glycosyl-phosphatidylinositol (GPI)-anchored lymphocyte proteins. II. GPI-anchored and transmembrane molecules internalize through distinct pathways Eur J Immunol 1992 22 15 21 10.1002/eji.1830220104 1346109

[B47] Bamezai A Rock KL Effect of ras-activation on the expression of glycosyl-phosphatidylinositol-anchored proteins on the plasma membrane Oncogene 1991 6 1445 1451 1679532

[B48] Keller GA Siegel MW Caras IW Endocytosis of glycophospholipid-anchored and transmembrane forms of CD4 by different endocytic pathways Embo J 1992 11 863 874 1532143 10.1002/j.1460-2075.1992.tb05124.x PMC556526

[B49] Deckert M Ticchioni M Bernard A Endocytosis of GPI-anchored proteins in human lymphocytes: role of glycolipid-based domains, actin cytoskeleton, and protein kinases J Cell Biol 1996 133 791 799 10.1083/jcb.133.4.791 8666664 PMC2120835

[B50] Mayor S Rothberg KG Maxfield FR Sequestration of GPI-anchored proteins in caveolae triggered by cross-linking Science 1994 264 1948 1951 10.1126/science.7516582 7516582

[B51] Brown DA Rose JK Sorting of GPI-anchored proteins to glycolipid-enriched membrane subdomains during transport to the apical cell surface Cell 1992 68 533 544 10.1016/0092-8674(92)90189-J 1531449

[B52] Mayor S Maxfield FR Insolubility and redistribution of GPI-anchored proteins at the cell surface after detergent treatment Mol Biol Cell 1995 6 929 944 7579703 10.1091/mbc.6.7.929 PMC301249

[B53] Kamen BA Wang MT Streckfuss AJ Peryea X Anderson RG Delivery of folates to the cytoplasm of MA104 cells is mediated by a surface membrane receptor that recycles J Biol Chem 1988 263 13602 13609 3417674

[B54] Rothberg KG Ying YS Kamen BA Anderson RG Cholesterol controls the clustering of the glycophospholipid-anchored membrane receptor for 5-methyltetrahydrofolate J Cell Biol 1990 111 2931 2938 10.1083/jcb.111.6.2931 2148564 PMC2116385

[B55] Fujimoto T GPI-anchored proteins, glycosphingolipids, and sphingomyelin are sequestered to caveolae only after crosslinking J Histochem Cytochem 1996 44 929 941 8756764 10.1177/44.8.8756764

[B56] Parton RG Joggerst B Simons K Regulated internalization of caveolae J Cell Biol 1994 127 1199 1215 10.1083/jcb.127.5.1199 7962085 PMC2120257

[B57] Schnitzer JE Oh P McIntosh DP Role of GTP hydrolysis in fission of caveolae directly from plasma membranes Science 1996 274 239 242 10.1126/science.274.5285.239 8824187

[B58] Mayor S Sabharanjak S Maxfield FR Cholesterol-dependent retention of GPI-anchored proteins in endosomes Embo J 1998 17 4626 4638 10.1093/emboj/17.16.4626 9707422 PMC1170792

[B59] Sabharanjak S Sharma P Parton RG Mayor S GPI-anchored proteins are delivered to recycling endosomes via a distinct cdc42-regulated, clathrin-independent pinocytic pathway Dev Cell 2002 2 411 423 10.1016/S1534-5807(02)00145-4 11970892

[B60] Varma R Mayor S GPI-anchored proteins are organized in submicron domains at the cell surface Nature 1998 394 798 801 10.1038/29563 9723621

[B61] Chen H Yang J Low PS Cheng JX Cholesterol level regulates endosome motility via Rab proteins Biophys J 2008 94 1508 1520 10.1529/biophysj.106.099366 17981910 PMC2212687

[B62] Chatterjee S Smith ER Hanada K Stevens VL Mayor S GPI-anchoring leads to sphingolipid-dependent retention of endocytosed proteins in the recycling endosomal compartment Embo J 2001 20 1583 1592 10.1093/emboj/20.7.1583 11285223 PMC145477

[B63] Schwerer B Lassmann H Kitz K Bernheimer H Ganglioside GM1, a molecular target for immunological and toxic attacks: similarity of neuropathological lesions induced by ganglioside-antiserum and cholera toxin Acta Neuropathol 1986 72 55 61 10.1007/BF00687947 3825507

[B64] Kirkham M Fujita A Chadda R Nixon SJ Kurzchalia TV Sharma DK Pagano RE Hancock JF Mayor S Parton RG Ultrastructural identification of uncoated caveolin-independent early endocytic vehicles J Cell Biol 2005 168 465 476 10.1083/jcb.200407078 15668297 PMC2171740

[B65] Chadda R Howes MT Plowman SJ Hancock JF Parton RG Mayor S Cholesterol-sensitive Cdc42 activation regulates actin polymerization for endocytosis via the GEEC pathway Traffic 2007 8 702 717 10.1111/j.1600-0854.2007.00565.x 17461795 PMC7617178

[B66] Blank N Schiller M Krienke S Wabnitz G Ho AD Lorenz HM Cholera toxin binds to lipid rafts but has a limited specificity for ganglioside GM1 Immunol Cell Biol 2007 85 378 382 10.1038/sj.icb.7100045 17325693

[B67] Radhakrishna H Klausner RD Donaldson JG Aluminum fluoride stimulates surface protrusions in cells overexpressing the ARF6 GTPase J Cell Biol 1996 134 935 947 10.1083/jcb.134.4.935 8769418 PMC2120964

[B68] Radhakrishna H Donaldson JG ADP-ribosylation factor 6 regulates a novel plasma membrane recycling pathway J Cell Biol 1997 139 49 61 10.1083/jcb.139.1.49 9314528 PMC2139810

[B69] Caplan S Naslavsky N Hartnell LM Lodge R Polishchuk RS Donaldson JG Bonifacino JS A tubular EHD1-containing compartment involved in the recycling of major histocompatibility complex class I molecules to the plasma membrane Embo J 2002 21 2557 2567 10.1093/emboj/21.11.2557 12032069 PMC126039

[B70] Naslavsky N Weigert R Donaldson JG Characterization of a nonclathrin endocytic pathway: membrane cargo and lipid requirements Mol Biol Cell 2004 15 3542 3552 10.1091/mbc.E04-02-0151 15146059 PMC491817

[B71] Kalia M Kumari S Chadda R Hill MM Parton RG Mayor S Arf6-independent GPI-anchored protein-enriched early endosomal compartments fuse with sorting endosomes via a Rab5/phosphatidylinositol-3'-kinase-dependent machinery Mol Biol Cell 2006 17 3689 3704 10.1091/mbc.E05-10-0980 16760436 PMC1525230

[B72] Pelkmans L Kartenbeck J Helenius A Caveolar endocytosis of simian virus 40 reveals a new two-step vesicular-transport pathway to the ER Nat Cell Biol 2001 3 473 483 10.1038/35074539 11331875

[B73] Szabo I Simon M Jr Hunyadi J Plasmin promotes keratinocyte migration and phagocytic-killing accompanied by suppression of cell proliferation which may facilitate re-epithelialization of wound beds Clin Dev Immunol 2004 11 233 240 10.1080/17402520400001710 15559369 PMC2486324

[B74] Li WY Chong SS Huang EY Tuan TL Plasminogen activator/plasmin system: a major player in wound healing? Wound Repair Regen 2003 11 239 247 10.1046/j.1524-475X.2003.11402.x 12846910

[B75] Czekay RP Kuemmel TA Orlando RA Farquhar MG Direct binding of occupied urokinase receptor (uPAR) to LDL receptor-related protein is required for endocytosis of uPAR and regulation of cell surface urokinase activity Mol Biol Cell 2001 12 1467 1479 11359936 10.1091/mbc.12.5.1467 PMC34598

[B76] Caiolfa VR Zamai M Malengo G Andolfo A Madsen CD Sutin J Digman MA Gratton E Blasi F Sidenius N Monomer dimer dynamics and distribution of GPI-anchored uPAR are determined by cell surface protein assemblies J Cell Biol 2007 179 1067 1082 10.1083/jcb.200702151 18056417 PMC2099195

[B77] Czekay RP Aertgeerts K Curriden SA Loskutoff DJ Plasminogen activator inhibitor-1 detaches cells from extracellular matrices by inactivating integrins J Cell Biol 2003 160 781 791 10.1083/jcb.200208117 12615913 PMC2173358

[B78] Tang CH Wei Y The urokinase receptor and integrins in cancer progression Cell Mol Life Sci 2008 65 1916 1932 10.1007/s00018-008-7573-9 18345479 PMC11131927

[B79] Wang XQ Sun P Paller AS Gangliosides inhibit urokinase-type plasminogen activator (uPA)-dependent squamous carcinoma cell migration by preventing uPA receptor/alphabeta integrin/epidermal growth factor receptor interactions J Invest Dermatol 2005 124 839 848 10.1111/j.0022-202X.2005.23669.x 15816844

[B80] Shyng SL Huber MT Harris DA A prion protein cycles between the cell surface and an endocytic compartment in cultured neuroblastoma cells J Biol Chem 1993 268 15922 15928 8101844

[B81] Shyng SL Heuser JE Harris DA A glycolipid-anchored prion protein is endocytosed via clathrin-coated pits J Cell Biol 1994 125 1239 1250 10.1083/jcb.125.6.1239 7911471 PMC2290925

[B82] Pauly PC Harris DA Copper stimulates endocytosis of the prion protein J Biol Chem 1998 273 33107 33110 10.1074/jbc.273.50.33107 9837873

[B83] Perera WS Hooper NM Ablation of the metal ion-induced endocytosis of the prion protein by disease-associated mutation of the octarepeat region Curr Biol 2001 11 519 523 10.1016/S0960-9822(01)00147-6 11413003

[B84] Magalhaes AC Silva JA Lee KS Martins VR Prado VF Ferguson SS Gomez MV Brentani RR Prado MA Endocytic intermediates involved with the intracellular trafficking of a fluorescent cellular prion protein J Biol Chem 2002 277 33311 33318 10.1074/jbc.M203661200 12070160

[B85] Martins VR Graner E Garcia-Abreu J de Souza SJ Mercadante AF Veiga SS Zanata SM Neto VM Brentani RR Complementary hydropathy identifies a cellular prion protein receptor Nat Med 1997 3 1376 1382 10.1038/nm1297-1376 9396608

[B86] Hooper NM Taylor DR Watt NT Mechanism of the metal-mediated endocytosis of the prion protein Biochem Soc Trans 2008 36 1272 1276 10.1042/BST0361272 19021539

[B87] Parkyn CJ Vermeulen EG Mootoosamy RC Sunyach C Jacobsen C Oxvig C Moestrup S Liu Q Bu G Jen A Morris RJ LRP1 controls biosynthetic and endocytic trafficking of neuronal prion protein J Cell Sci 2008 121 773 783 10.1242/jcs.021816 18285446

[B88] Taylor DR Hooper NM The low-density lipoprotein receptor-related protein 1 (LRP1) mediates the endocytosis of the cellular prion protein Biochem J 2007 402 17 23 10.1042/BJ20061736 17155929 PMC1783995

[B89] Kaneko K Vey M Scott M Pilkuhn S Cohen FE Prusiner SB COOH-terminal sequence of the cellular prion protein directs subcellular trafficking and controls conversion into the scrapie isoform Proc Natl Acad Sci USA 1997 94 2333 2338 10.1073/pnas.94.6.2333 9122195 PMC20088

[B90] Hornemann S Glockshuber R A scrapie-like unfolding intermediate of the prion protein domain PrP(121-231) induced by acidic pH Proc Natl Acad Sci USA 1998 95 6010 6014 10.1073/pnas.95.11.6010 9600908 PMC27576

[B91] Zanusso G Farinazzo A Fiorini M Gelati M Castagna A Righetti PG Rizzuto N Monaco S pH-dependent prion protein conformation in classical Creutzfeldt-Jakob disease J Biol Chem 2001 276 40377 40380 10.1074/jbc.C100458200 11682490

[B92] Fivaz M Vilbois F Thurnheer S Pasquali C Abrami L Bickel PE Parton RG Goot FG van der Differential sorting and fate of endocytosed GPI-anchored proteins Embo J 2002 21 3989 4000 10.1093/emboj/cdf398 12145200 PMC126144

[B93] Pagano RE Endocytic trafficking of glycosphingolipids in sphingolipid storage diseases Philos Trans R Soc Lond B Biol Sci 2003 358 885 891 10.1098/rstb.2003.1275 12803922 PMC1693187

[B94] Lundmark R Doherty GJ Howes MT Cortese K Vallis Y Parton RG McMahon HT The GTPase-activating protein GRAF1 regulates the CLIC/GEEC endocytic pathway Curr Biol 2008 18 1802 1808 10.1016/j.cub.2008.10.044 19036340 PMC2726289

[B95] Chang WJ Rothberg KG Kamen BA Anderson RG Lowering the cholesterol content of MA104 cells inhibits receptor-mediated transport of folate J Cell Biol 1992 118 63 69 10.1083/jcb.118.1.63 1618907 PMC2289528

[B96] Nichols BJ GM1-containing lipid rafts are depleted within clathrin-coated pits Curr Biol 2003 13 686 690 10.1016/S0960-9822(03)00209-4 12699627

